# Genome-wide survey of miRNAs and their evolutionary history in the ascidian, *Halocynthia roretzi*

**DOI:** 10.1186/s12864-017-3707-5

**Published:** 2017-04-20

**Authors:** Kai Wang, Christelle Dantec, Patrick Lemaire, Takeshi A. Onuma, Hiroki Nishida

**Affiliations:** 1 Department of Biological Sciences, Graduate School of Science, Osaka University, Toyonaka, Osaka 560-0043 Japan; 2 Centre de Recherches de Biologie cellulaire de Montpellier (CRBM), UMR5237, CNRS-Université de Montpellier, 1919 route de Mende, F-34090 Montpellier, France; 3 Present address: Shanghai Key Laboratory of Molecular Andrology, Institute of Biochemistry and Cell Biology, Shanghai Institute of Biological Science, Chinese Academy of Sciences, 320 Yue Yang Road, Shanghai, 200031 People’s Republic of China

**Keywords:** Ascidian, *Halocynthia roretzi*, miRNA, Genome-wide survey, miRNA targets

## Abstract

**Background:**

miRNAs play essential roles in the modulation of cellular functions via degradation and/or translation attenuation of target mRNAs. They have been surveyed in a single ascidian genus, *Ciona*. Recently, an annotated draft genome sequence for a distantly related ascidian, *Halocynthia roretzi*, has become available, but miRNAs in *H. roretzi* have not been previously studied.

**Results:**

We report the prediction of 319 candidate *H. roretzi* miRNAs, obtained through three complementary methods. Experimental validation suggests that more than half of these candidate miRNAs are expressed during embryogenesis. The majority of predicted *H. roretzi* miRNAs appear specific to ascidians or tunicates, and only 32 candidates, belonging to 25 families, are widely conserved across metazoans.

**Conclusion:**

Our study presents a comprehensive identification of candidate *H. roretzi* miRNAs. This resource will facilitate the study of the mechanisms for miRNA-controlled gene regulatory networks during ascidian development. Further, our analysis suggests that the majority of *Halocynthia* miRNAs are specific to ascidian or tunicates, with only a small number of widely conserved miRNAs. This result is consistent with the general notion that animal miRNAs are less conserved between taxa than plant ones.

**Electronic supplementary material:**

The online version of this article (doi:10.1186/s12864-017-3707-5) contains supplementary material, which is available to authorized users.

## Background

miRNAs are a class of short endogenous non-coding regulatory RNAs whose length is approximately 22 nt. They modulate various biological processes, such as cellular differentiation, proliferation, apoptosis, development and homeostasis [1–4]. They act by repressing translation or destabilizing target mRNA, thereby providing an additional layer of control in gene regulatory networks [5]. In animals, a seed sequence is present at nucleotides 2–7 of the mature sequence and is a major determinant of miRNA targeting specificity. miRNAs sharing the same seed are considered to belong to the same family [6]. miRNA genes generally locate in non-coding intergenic or intronic regions [7], with some rare cases found in protein-coding regions [8]. The activity of miRNA genes is often restricted to specific developmental stages or tissues, and their expression is sometimes only stimulated by environmental cues such as temperature [9, 10], oxidative [11], salt or drought [12] stresses. While high-throughput small RNA sequencing (miRNA-seq) [13–16] provides a powerful approach for miRNA identification, their restricted expression makes it difficult to use this method to exhaustively survey miRNAs in a given species. Potential miRNA genes can also be computationally predicted in whole genome sequences, and this could usefully complement miRNA-seq approaches.

Ascidians (Phylum: Chordata, Subphylum: Tunicata, Class: Ascidiacea) have been used as model species in development biology for over a century [17, 18]. These species offer attractive experimental features, including a compact genome (e.g. *Halocynthia roretzi* genome is around 170 Mb with about 16,000 protein-coding genes [19]), invariant embryonic cell lineages, small embryonic cell number, and translucent embryos, which allow the description of developmental processes with a cellular level of resolution. 15 years ago, the complete genome sequences of two ascidian species, *Ciona robusta* [20] (formerly *Ciona intestinalis* Type A [21]) and *Ciona savignyi* [22] were assembled, annotated and made publicly accessible through genome browsers [19]. Since then, the genomes of additional tunicate species have been sequenced, partially annotated and publicly released [19, 23–26], opening the way to a study of the evolution of ascidian coding and non-coding genetic elements. It is generally considered that ascidians are subject to rapid molecular evolution, in both coding and non-coding sequences [27, 28].

Recently, many miRNAs have been described in *C. robusta* and *C. savignyi* (Order: Phlebobranchia) [29–33]. Over 400 miRNA candidates were predicted [31] and the expression of 380 of them was experimentally detected in *C. robusta* by miRNA-seq and microarray data [31, 32]. Some *C. robusta* miRNAs control development processes [19]. For example, miR-124 promotes neuronal development via the inhibition of Notch signaling [34, 35], while miR-1 and miR-133 have muscle-specific functions, as in vertebrates.

In this study, we performed a comprehensive search for miRNA in *H. roretzi*, an ascidian of a different order, the stolidobranchia, using a recently sequenced and annotated genome draft [19]. Three major approaches were used to predict miRNAs: conservation to miRNA described in miRBase, *de novo* miRNAs prediction, and similarity to *Ciona* small RNA-seq reads. A total of 319 miRNA genes were discovered, whose evolutionary conservation was studied. This study thus advances our understanding of the complex gene regulatory network of ascidian embryos and will facilitate future developmental biology studies.

## Result

### 61 miRBase metazoan miRNAs are conserved in *Halocynthia* and approximately half of them may be ascidian or tunicate-specific

**Fig. 1:**
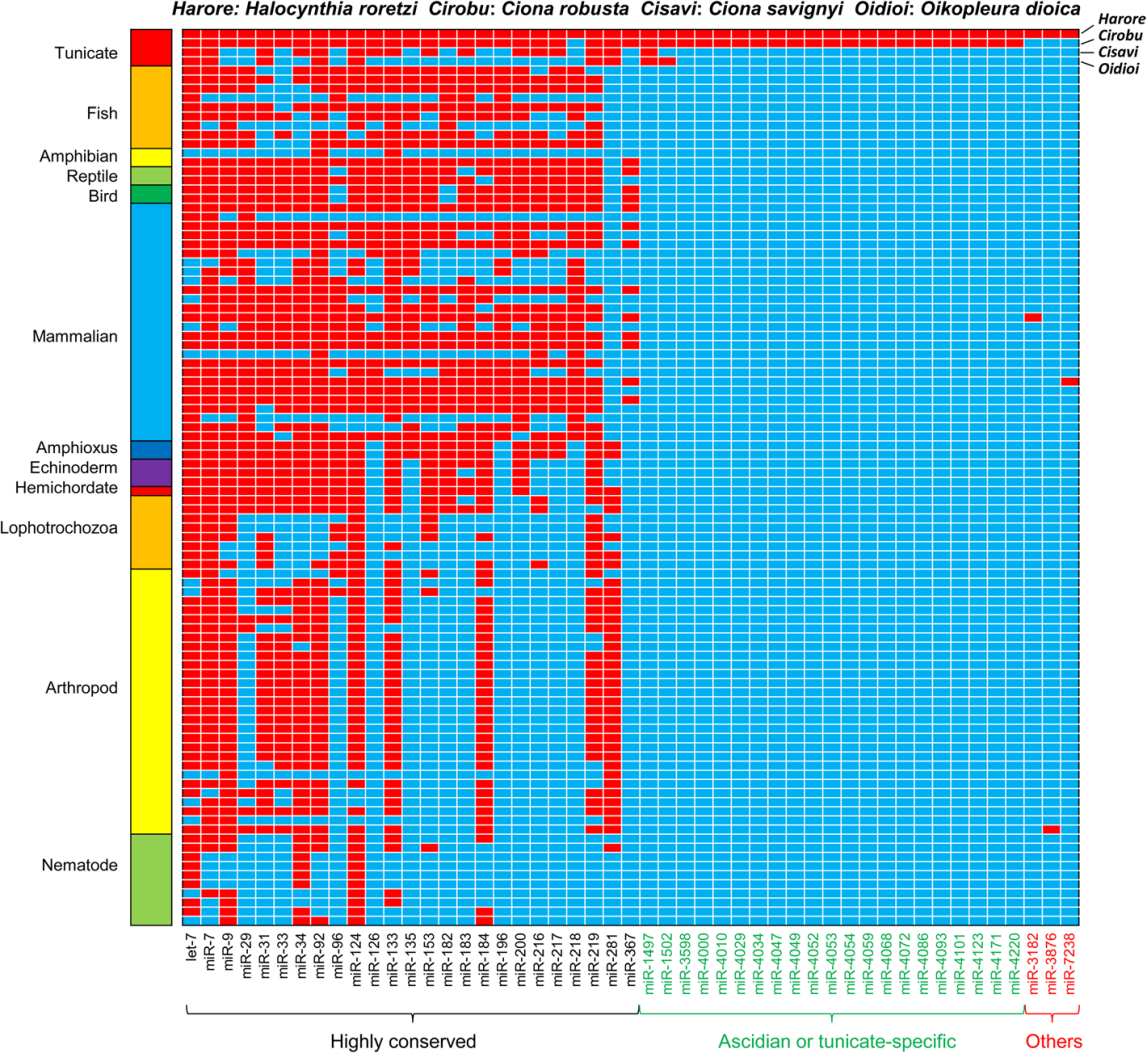
Phylogenic survey of the conserved *H. roretzi* miRNAs in other species. *Red squares* indicate that the miRNA exists in the corresponding species, *Light blue square* indicates that the miRNAs has not been reported in the species. 13 highly conserved families (miR-1, miR-15, miR-78, miR-101, miR-125, miR-132, miR-141 (miR-8), miR-155, miR-181, miR-199, miR-375, miR-672 and miR-1473) that were detected in *C. robusta* were not found in *H. roretzi* via our method. These miRNAs may exist in *H. roretzi*, without satisfying the stringent filtering criteria we applied. Alternatively, they may have been lost in the *H. roretzi* lineage. See details of species names in Additional file 2

**Fig. 2:**
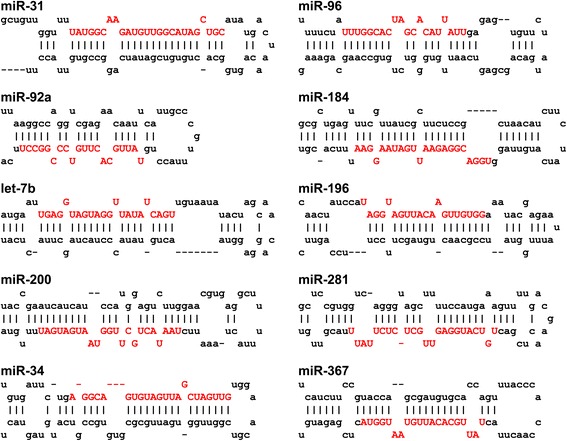
Stem-loop structure of some conserved known miRNA genes of *H. roretzi*. *Red upper-case* letters indicate mature miRNA sequences

To survey the repertoire of miRNAs in *H. roretzi*, we first carried out a BLASTN similarity search (word size 7, *E*-value <10, see Methods) in the *H. roretzi* genome, using as input all known mature metazoan miRNAs deposited in miRBase (28,645 entries) [36]. We further selected miRNA candidates whose flanking genome sequences passed our filtration criteria on the stem-loop structure and minimum folding free energy (MFE) (see Methods section for details). This identified 61 candidate *H. roretzi* miRNA precursors, belonging to 49 known miRNA families (Fig. 1, Table 1 and Additional file 1). Figure 2 shows the stem loops formed by genomic sequences flanking a selection of predicted *H. roretzi* miRNAs.

**Table 1:** Conserved miRNAs in *H. roretzi*

Location in Scaffolds (S) and the orientation (+,-)	Family	Mature sequences (5′-3′)	Precursor Length (nt)	MFE (kcal/mol)
S648_2373-2295:-	let-7	UGAGGUAGUAGGUUGUAUAGUUU	79	-30.4
S93_67805-67726:-	let-7	UGAGGUAGUGGAUUAUGCAGUU	80	-30.1
S93_68361-68283:-	let-7	UGAGGUAGUAGGUUAUAUCAGU	79	-22.6
S93_68526-68445:-	let-7	UGAGGUAGUAGGUUAUGUAGUG	82	-24.6
S93_68698-68609:-	let-7	CUGAGGUAGUAGGUUAUGCAGUU	90	-31.2
S24_284667-284752:+	miR-7	UGGAAGACUAGUGAUUUUGUUGUUC	86	-19.5
S159_72225-72288:+	miR-9	UCUUUGGUUAUCUAGUUUUGUG	64	-20.3
S93_128856-128791:-	miR-29	UAGCACCAUUGGAAAUCGGUC	66	-19.8
S65_9585-9666:+	miR-31	UAUGGCAAGAUGUUGGCAUAGCUGC	82	-34
S298_83663-83598:-	miR-33	AAGUGCAUUGUAGUUGCAUUGCACA	66	-20.8
S160_170587-170662:+	miR-34	AGGCAGUGUAGUUAGCUAGUUG	76	-19.3
S27_318389-318451:+	miR-92	UAUUGCACUUGUCCCGGCCU	63	-18.8
S57_155850-155929:+	miR-92	UAUUGCACUCGUCCCGGUCUAU	80	-21.5
S181_161093-161016:-	miR-96	UUUGGCACUAGCACAUUAUU	78	-25.2
S375_52432-52351:-	miR-124	CGUGUUCACUGCAGACCUU, CAUUAAGGCACGCGGUGAAUGCUAU	82	-30
S375_52641-52557:-	miR-124	AAUUAAGGCACGCGGUGAAUGCCAGA	85	-37.6
S375_60909-60992:+	miR-124	UAUUAAGGCACGCGGUGAAUGCCAAG	84	-37
S60_145419-145497:+	miR-126	CCUUGUUACUUACGGUACC, AUCUCGUACCGUGAGUAAUAAAGCU	79	-38.2
S6_131727-131818:+	miR-133	GCUGGUCAACCGGAACCAAAUC, UUUGGUCCCCUUCAACCAGCUGUU	92	-29.5
S56_118756-118667:-	miR-135	UAUGGCUUUAUUUAUUCCUGUGUGA	90	-32.9
S67_157861-157774:-	miR-153	GUCAUUUUUGUAUUAUGCAA, UUGCAUAGUAACAAAAGUGAUCAU	88	-40.3
S181_160894-160804:-	miR-182	CUUGGCAAAAUAUAGAACUC	91	-36.1
S181_165741-165631:-	miR-183	UAUGGCACUAGUAGAAUUCACUGC	111	-36.3
S602_41811-41890:+	miR-184	UGGACGGAGAAUUGAUAAGGAA	80	-30.4
S54_165680-165756:+	miR-196	UAGGUAGUUACAAGUUGUGG	77	-25.6
S353_78598-78518:-	miR-200	UAAUACUGCUUGGUAAUGAUGAU	81	-24.5
S244_68891-68803:-	miR-216	UAAUCUCAGCUGGCAAUCUGUGA	89	-35.3
S244_68624-68517:-	miR-217	AUACUGCAUUAGGAACUGAUUGGU	108	-30.3
S248_20812-20705:-	miR-218	UAUGUGCUUUGAUCUAACCAUGU	108	-34.9
S21_373535-373467:-	miR-219	UGAUUGUCCAAACGCAAUUCGCG	69	-19.3
S11_93983-94061:+	miR-281	UGUCAUGGAGUUGCUCUCUUAUU	79	-24.3
S99_176620-176695:+	miR-367	UAUUGCACAUUGUAAUGGUA	76	-29.3
S291_113322-113388:+	miR-1497	UUGAAGAAUUGCAGGUGGUAGGU	67	-23.2
S290_113331-113406:+	miR-1502	UUGAACUUUCUAAGGAAUAG	76	-30.2
S19_110094-110150:+	miR-3182	GCUUUUGUAGUUUAGUC	57	-20.4
S210_48343-48421:+	miR-3598	UCACAGUGGUUGUAUACUGC	79	-42.1
S176_129704-129781:+	miR-3876	GUUUUGUUUUAACACUUAC	78	-22.4

In the cases of miR-124, 126, 133, and 153, both of 5′ and 3′ mature sequences are reported in different species

The phylogenetic distribution of these *H. roretzi* 49 miRNA families in miRBase was next examined (Fig. 1 and Additional file 2). 25 families were highly conserved across metazoa, including let-7 and miR-7 to -367 (Fig. 1). Of these, 18 families were found in both deuterostomes and protostomes and may thus represent ancestral metazoan miRNAs. Seven families were exclusively found in deuterostomes, in either only chordates (6) or in both chordates and ambulacraria. We attribute the absence of miR-218 from *Ciona* and *Oikopleura* to the possible restricted expression of this miRNA, which may have precluded its identification by miRNA-seq. Interestingly, an ancestral metazoan miRNA, miR-281, appears to have been specifically lost from the vertebrate lineage, as it is present in all surveyed tunicates, amphioxus, and protostomes. The loss in echinoderms is not clear, since the number of species is only three.

Twenty one families were found in the distantly related *C. robusta* and *H. roretzi* ascidians but not in other animals (labeled in green on Fig. 1), and may thus correspond to ascidian or tunicate-specific miRNAs. These miRNAs are all represented by at least 10 reads in the *Ciona* small RNA sequencing dataset [32] (BLASTN, word size of 15, and E-value ≤1000), suggesting that they are expressed during *C. robusta* embryogenesis and are therefore likely to be genuine miRNAs. Finally, three miRNA families, miR-3182, miR-3876 and miR-7238 were only found in *H. roretzi* and a single non-tunicate species (Fig. 1, right most three columns). Our confidence in the predictions of these miRNAs is more limited.

To test whether the small number of candidate *Halocynthia* miRNAs conserved across metazoa reflected a low sensitivity in our identification method, or the overall weak conservation of ascidian miRNAs, we checked the situation in *C. robusta*. A total of 348 *C. robusta* miRNAs, belonging to 285 families, were previously identified and deposited in miRBase. These miRNAs were predicted from miRNA-seq data collected from *Ciona* embryos at the gastrula and larval stages, using the miRTRAP computational program [32], a method that makes no hypothesis on the evolutionary conservation of these candidates. Of these 348 miRNAs, only 47 miRNAs, belonging to 36 families, were widely conserved in many non-tunicate metazoan species (Additional file 3). An additional six miRNAs (*Cirobu*-mir-1473, *Cirobu*-mir-1497, *Cirobu*-mir-1502a, *Cirobu*-mir-1502b, *Cirobu*-mir-1502c, *Cirobu*-mir-1502d) belonging to three families were found in at least one tunicate species other than *C. robusta* (miRNA data had so far been described in three tunicate species, *C. robusta* [29–32], *Ciona savignyi*, 43 miRNAs in miRBase [32, 36, 37], and *Oikopleura dioica*, 69 miRNAs reported, [36, 37]). miRNA candidate *Cirobu*-mir-3575 was also found in *Rattus norvegicus*. The remaining 294 *C. robusta* candidate miRNAs (belonging to 239 families) appeared to be specific for *C. robusta*.

The evolutionary analysis of these *Ciona* miRNAs, and the small number of *Halocynthia* candidate miRNA detected by conservation to miRBase entries suggest that a majority of ascidian miRNAs may be either ascidian or tunicate-specific. Discriminating between these two possibilities is currently difficult as the number of miRNA reported so far in the non-ascidian tunicate *Oikopleura dioica* (*n* = 69) are small, suggesting that the list could be far from complete. Similarly, the current repertoire of *Ciona savignyi* miRNA (*n* = 43) is incomplete, explaining the small number of the miRNAs for this species listed in Fig. 1.

### *De novo* miRNAs prediction

**Fig. 3:**
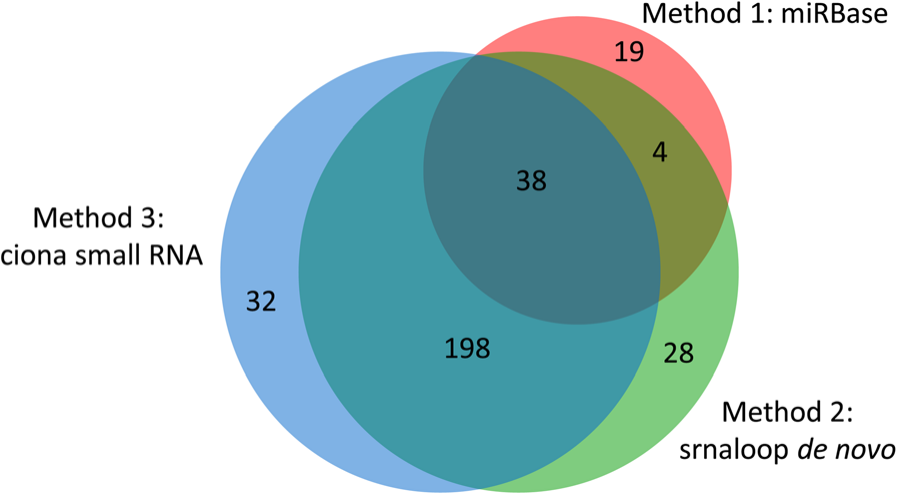
Numbers of miRNAs predicted by three different methods. Numbers of miRNAs that were found in this study are presented in the Venn diagram. There is significant overlap between miRNAs that were found by the three kinds of approaches

To extend our study of the *Halocynthia* miRNA repertoire, we next used srnaloop [38], to detect potential miRNA precursors on the sole basis of the presence of a canonical stem-loop structure [16, 39]. Genomic sequences that met minimum folding free energy (MFE) and stem-loop structure filtration criteria were considered as potential novel miRNA precursors. Clustering of these candidate sequences using CD-HIT (sequence identity threshold: 0.9) [40] identified some clusters with high similarity. Some of these clustered sequences were mapped to repeated sequences, which could form palindromes and confound srnaloop. These sequences were excluded from the final predictions. This approach predicted 268 miRNA candidates including 42 that were found in the previous section (Fig. 3, Additional file 1, miR-5000 to -5257). These novel candidates showed no significant hit by BLASTX (E-value ≤ 1e-3) with proteins in nr and UniProtKB/Swiss-Prot database confirming that they are non-coding RNAs.

**Fig. 4:**
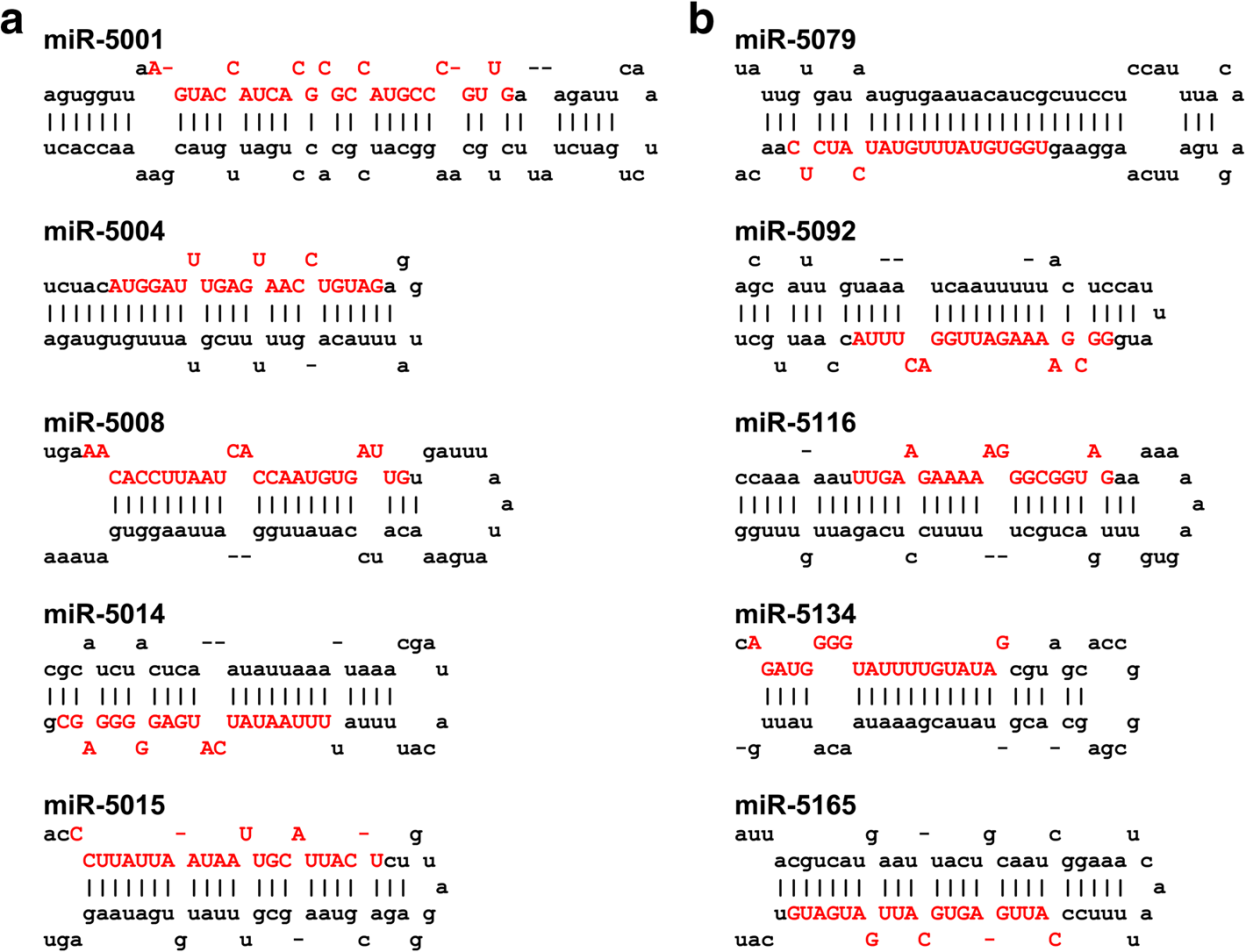
Stem-loop structure of some novel miRNAs genes. **a** miRNAs detected with high abundance (≥50 reads) in *C. robusta* small RNA-Seq data. **b** miRNAs observed with low abundance (≤10 reads) in small RNA-Seq data. *Red upper-case* letters indicate inferred mature miRNA sequences

miRNA precursors are transcribed from only one of the double-stranded DNA template. Which strand the miRNA precursors originated from was inferred in 291 cases from published transcript information (see Method section). Prediction of the mature miRNA sequences for *de novo* predicted miRNAs is difficult and moderately reliable as the precise mechanism by which Dicer cuts miRNAs from the hairpin structure remains unknown. Li *et al* [7] reported an inference method for mature miRNA locations by supposing that Dicer precisely cuts the mature miRNA at the loop-stem junction. We note, however, that the majority of miRBase miRNAs do not obey this rule. Furthermore, 5p and 3p miRNAs are localized to the two parallel arms of the hairpin structure, and are not exactly complementary in sequence (see Fig. 2). So we tentatively predict the positions of 5p and 3p miRNAs by detecting paired segments using patscan although we do not know which of 5p and/or 3p is the actual miRNA [13, 41, 42]. For those precursors, in which patscan failed to find the paired sequences, a modified Li’s method was used to find the 5p and 3p segments. The sequence of predicted mature miRNAs are presented in Fig. 4a and b.

### Analysis of *C. robusta* small RNA-Seq data confirms *de novo* miRNA predictions

As the first test of our *de novo* predictions of *Halocynthia* miRNAs, we next made use of a previously generated set of *C. robusta* small RNA-Seq reads [32]. These reads, which are enriched in miRNAs, but could also include other classes of small non-coding RNAs [43], were mapped onto the *H. roretzi* reference genome using BLASTN (see Methods), and hits flanked by sequences whose minimum folding free energy (MFE) and stem-loop structure passed our filtration criteria were selected for further analysis (see Methods section for details). This identified 268 novel candidate miRNAs. Remarkably this set included 236 of the 268 candidate miRNA predicted by our *de novo* approach, of which 38 were also detected using miRBase data (Fig. 3; Additional file 1; ID numbers of the novel *H. roretzi* miRNAs that were detected in these ways are from 5000 to 5229). Figure 4a shows the stem-loop structures of a selection of these novel miRNA genes, with high small RNA-Seq reads (≥50 reads) support. Figure 4b showed stem-loop structures of miRNA genes with weak small RNA-Seq reads (≤10 reads) support, which represents low confidence predictions. The mature sequences of these *H. roretzi* miRNAs were inferred via the matched position of *C. robusta* small RNA-Seq reads (Fig. 4, red letters).

Interestingly mature miRNA sequences appear to have diverged significantly between *Ciona* and *Halocynthia*. Of the 230 novel predicted mature *H. roretzi* miRNAs (268 hits minus 38 identified in Fig. 3), *Hrore*-miR-5008 was the only one with less than two mismatches to the 458 *C. robusta* mature miRNAs predicted by Keshavan et al. [31]. When four mismatches were allowed, only five hits were detected, *Harore*-miR-5008 (*Cirobu-*miR-13a), *Harore*-miR-5046 (*Cirobu-*miR-200b), *Harore*-miR-5154_5p (*Cirobu-*miR-244), *Harore*-miR-5165 (*Cirobu-*miR-246), and *Harore*-miR-5214 (*Cirobu-*miR-246).

In addition to 458 *C. robusta* mature miRNAs, we reexamined whether the 230 novel *Halocynthia* miRNAs have homologues in the entire *C. robusta* genome (http://ghost.zool.kyoto-u.ac.jp/download_kh.html). When two mismatches were allowed in the mature sequences, 168 *H. roretzi* miRNAs were found to have a homolog in the *C. robusta* genome (BLASTN top 500 hits, word size of 7, and an alignment length of ≥20). When four mismatches were accepted, however, 225 out of 230 (97.8%) *H. roretzi* miRNAs were found to have a homolog in the *C. robusta* genome and 66 (29.3%) of these 225 precursors could form canonical stem-loops when the temperature parameters of RNAfold were adjusted to 18 °C [31] (Additional file 1, right most column). We have analyzed positions of the mismatches. 24.5% occurred in seed region (base 2–7) and 75.5% occurred outside of the seed region, suggesting that mismatches distribute evenly over entire miRNAs as base number of seed region is only six out of 20–24 nucleotide. These results suggest that, although the sequences of mature ascidian miRNAs may diverge rapidly, *C. robusta* and *H. roretzi* may share more homologous miRNAs than expected from the results of the previous section.

The union of the miRNAs predicted by all approaches consists of 319 candidate miRNAs in the *Halocynthia* genome: 61 well-conserved miRNAs, 226 *de novo* predicted miRNAs and 32 additionally predicted from *Ciona* small RNA-Seq data. These predictions largely overlap (Fig. 3).

### Validation of potential miRNAs prediction

**Fig. 5:**
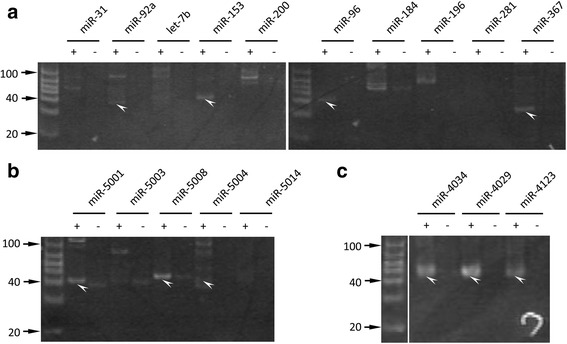
RT-PCR validation of miRNAs. **a** RT-PCR results of some well-conserved miRNAs listed in Table 1. **b** RT-PCR validation of some potential miRNAs that were predicted *de novo* and by using *C. robusta* small RNA-Seq reads. **c** Ascidian or tunicate-specific miRNAs (miR-4034, 4029 and 4123)*.* ‘ + ’ indicates PCR with reverse transcriptase; ‘-’ indicates PCR without reverse transcriptase. The expected size of the amplified fragment is ~40 bp (*arrowheads*) because the 19 bp universal primer is amplified together with ~20 bp miRNA-specific primers. Other bands would be non-specific bands

Expression of some of the predicted miRNAs was validated by RT-PCR using embryonic RNA. The primers used are listed in Additional file 4. RNA from mixed stages of embryogenesis was purified. In total, 20 miRNA candidates were tested, and bands of adequate size were detected for 10 miRNAs (Fig. 5). Sequencing of these 10 PCR bands confirmed their identity. No bands were amplified in RT negative samples. In the 10 well-conserved miRNAs (Table 1; most of them are also shown in Fig. 2), miR-92a, miR-153, miR-96 and miR-367 showed clear bands of ~40 bp (Fig. 5a). We also examined 5 potential miRNAs that were predicted *de novo* and from *C. robusta* small RNA-Seq reads (Fig. 5b, some of them are shown in Fig. 4a). miR-5001, miR-5004 and miR-5008 showed a clear band at ~40 bp. In addition, we found that the faint bands of miR-5003 and 5014 in Fig. 5b also contained the expected sequences. Three miRNAs in miRBase that are conserved only between *H. roretzi* and *C. robusta* (miR-4034, miR-4029 and miR-4123) gave clear bands (Fig. 5c). These results indicate the embryonic expression of at least half of predicted miRNAs. The other predicted miRNAs may either correspond to artefactual prediction, or not have been amplified because the position of their predicted mature sequence may be erroneous resulting in inadequate primer choice, or because these miRNAs are not expressed during embryogenesis.

### Potential target prediction of the miRNAs

To get insight into the functions of the miRNAs, 3′ UTR sequences were extracted from each gene model in Aniseed [19] (http://www.aniseed.cnrs.fr/). Then, targets were tentatively predicted. A total of 3451 possible target sites in putative 3′ UTR sequences, which correspond to 17% of coding genes (2734 genes out of approximately 16,000 gene models), were detected for 275 miRNAs. Among those, 285 target genes of 140 miRNAs have gene ontology (GO) terms associated with various development processes. Although the functional validation of these targets goes beyond the scope of this article and these targets have not been functionally validated, information on their identity, provided as a list in Additional file 5, may be useful for future studies of miRNA functions.

## Discussion

Compared to high-throughput small RNA sequencing, computational miRNA discovery approaches offer several advantages when reference genome sequence is available. First, they do not need the availability of small RNA-Seq. Second, it could in theory discover all possible miRNAs, while small RNA sequencing can only identify miRNAs expressed in the cells, tissues, organs, from which the RNA was collected. This is particularly useful as some miRNAs are only expressed in response to stresses, such as hyper-salinity, hyper osmotic pressure and disease. On the other hand, the disadvantage of computational predictions is that no direct experimental support of the predicted miRNAs is provided until their expression is validated via RT-PCR or small RNA-Seq.

In this study, the repertoire of miRNAs in the *H. roretzi* genome was investigated by bioinformatics methods (Fig. 3 and Additional file 1) using three methods: homology search using mature miRNAs sequences deposited in miRBase (method 1 in Fig. 3), *de novo* miRNAs prediction using srnaloop (method 2), and prediction based on sequence similarity with *C. robusta* small RNA-Seq data (method 3). We found 61 conserved miRNAs, 226 additional miRNAs predicted by srnaloop, and another non-overlapping set of 32 miRNAs using *C. robusta* small RNA-Seq data. 38 conserved miRNAs were predicted by all three methods, a significant overlap supporting the reliability of the methods used in the present study.

**Fig. 6:**
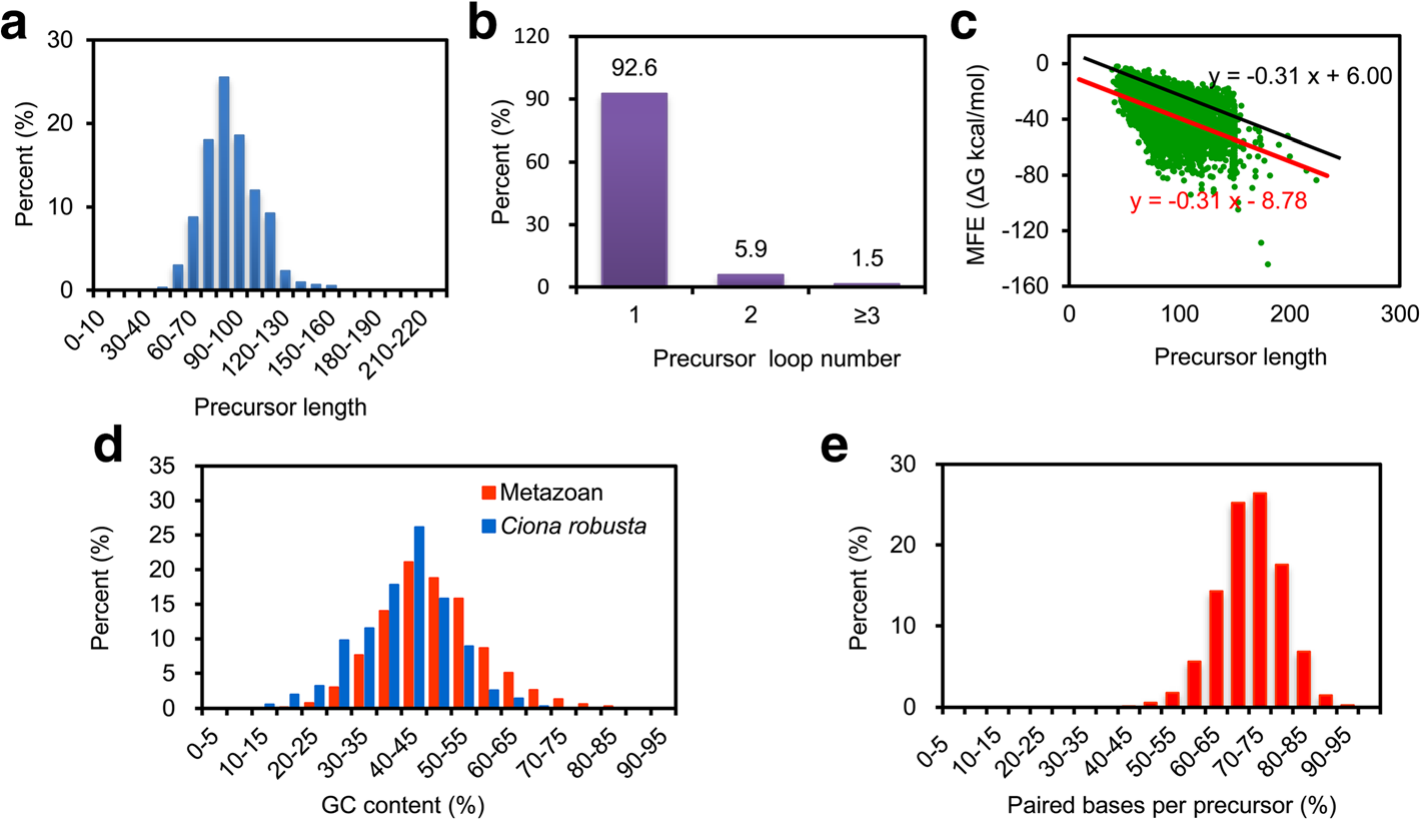
Criterions that were used in this study and its evaluation by application to metazoan miRNA precursors. **a** Precursor length distribution and their percent of metazoan miRNA precursors that are registered in miRBase. **b** Loop number per precursor. Loop number was predicted by RNAfold. **c** Relationship of MFE and precursor length. MFE was calculated by RNAfold. *Red line* indicates the equation simulated via linear regression analysis. *Black line* represents the equation with the shifted constant used in this study, and it allowed ~95.3% precursors to pass the criterion, MFE ≤ -0.31 * L + 6.00. **d** GC content distribution. **e** Paired bases per precursor. Paired bases per precursor were obtained by RNAfold results

These miRNAs sum up to 319 in total, a lower number than described in *Ciona* [31], suggesting that our repertoire may be incomplete. To estimate the proportion of undiscovered miRNAs, we investigated how many metazoan miRNA precursors deposited in the miRBase would pass our selection criteria used in this study in terms of precursor length, loop number, MFE, GC content, and precursor base pairing situation as shown below. Nucleotide lengths of 97.4% precursors are between 50 and 130 bp (Fig. 6a), 92.6% precursors have only one loop (Fig. 6b), 95.3% precursors’ minimum folding free energy (MFE) ≤ -0.31 * L + 6.00 (Fig. 6c), 94.7% precursors’ GC content are between 30 and 70% (Fig. 6d), 97.5% precursors have more than 55% paired bases (Fig. 6e). Despite the individual recovery rate are pretty high, however, only 67.8% miRNAs passed all of these criteria. Therefore, roughly 32% of miRNAs could not pass our criteria and still to be discovered in *H. roretzi*.

Thirty two (25 families) of 319 miRNAs (10%) are well-conserved miRNAs over several phyla, and 24 (21 families) are shared only with *C. robusta*. In addition, 230 novel miRNAs of *H. roretzi* were predicted. 41 of these may correspond to genuine miRNAs in *C. robusta*. Therefore, 20% (65) miRNAs may correspond to ascidian/tunicate-specific miRNAs shared between *H. roretzi* (order Stolidobranchia) and *C. robusta* (order Stolidobranchia), although this estimation is still rough. The situation is similar in *C. robusta.* We checked the 348 previously identified miRNAs in *C. robusta* that are deposited in miRBase [44] and found that only 47 of them are well-conserved miRNAs over phyla. Therefore, it seems that ascidian species have small number of widely conserved miRNAs, and a larger number of ascidian/tunicate-specific miRNAs. These results are consistent with the general conjecture that animal miRNAs are not well conserved between distant taxa [45].

## Conclusion

miRNAs play crucial roles in the modulation of developmental processes as well as response to environment stresses, but little is known about their functions in the tunicate species. We reported potential *H. roretzi* miRNAs inferred from the genome sequence, and showed that only a small number of miRNAs were conserved across phyla. Most miRNAs were newly discovered in this study. Our study suggested the possibility that many miRNAs could be conserved among ascidian species. This finding would hopefully facilitate future studies of gene regulation by miRNAs.

## Methods

### Data Preparation & Pre-processing

The *H. roretzi* genome sequences were assembled by us and are publicly available through the Aniseed database (http://www.aniseed.cnrs.fr/) [19]. Prior to miRNA predication, we masked genomic regions with less miRNA-cording possibility. This includes protein coding and non-coding RNA generating regions that are rRNAs, tRNAs, snoRNAs and lncRNAs. To be specific, coding sequences and repeat regions were masked firstly. Coding regions were obtained from the predicted gene models in the Aniseed, and repeat regions were generated by RepeatMasker and RepeatModeler (http://repeatmasker.org). Tandem repeats were masked by Tandem Repeats Finder [46]. Other RNAs, including rRNAs, tRNAs, snRNAs and lncRNAs were filtered using Rfam database (Release 11.0) [47] by cmsearch in INFERNAL [48] (E-value threshold: 1e-3). Potential tRNAs were also screened by tRNAscan-SE [49].

### Identification of widely conserved miRNAs and precursors

To reduce the search space, we firstly queried all metazoan mature miRNA seeds deposited in miRBase database (Release 21) [44] against the masked *H. roretzi* genome sequences using blastn with a word size of 7 and E-value of 10. The 110 bp sequences of matched genomic regions were retrieved separately and extended for additional 20 bp from both 5′ and 3′ ends. Potential miRNAs with no more than two mismatches with known metazoan miRNAs were identified by patscan [13, 41, 42], and the candidates were folded by RNAfold [50, 51] subsequently. Unmatched sequences were trimmed. If more than one miRNA were mapped to the overlapping position, only the best one with the minimum folding free energy (MFE) was kept. In addition, Rfam (Release 11.0) [52] covariance model (CM) was also used to search for conserved miRNA structures. Low complexity sequences were removed. We retained sequences whose minimum folding free energy (MFE) and stem-loop structure passed our filtration criteria that are mentioned below.

### *De novo* miRNAs prediction

To detect potential miRNA precursors, srnaloop [38] was employed to identify hairpin structures from masked *H. roretzi* genome sequence. We used the parameter “-st d -Gs -1.5 -gu 1 -sml 100000000 -am 990000 -t 15 -lml 5”. “-st d” means that we used the DNA sequences, “-Gs -1.5” means that gap start score is -1.5, “-gu 1” means that GU base pairs score is 1, “-t 15” means that alignment score threshold is 15, “-lml 5” means that minimum size of hairpin loop is 5. The sequence length parameter “-sml” and the maximum hairpin alignments “-am” parameters were set at their maximal possible value (100000000 and 900000). In srnaloop software, the predicated hairpin lengths were less than and tend to be close to the length parameter that is set, while the miRNA precursors length generally ranges from 60 to120 (Fig. 6a). Therefore, the maximum length of hairpin sequence parameter “-l” was set from 60 to 130 with an interval of 10, and chose the shortest hairpin that passed the folding free energy evaluation. Potential precursors were further blasted against nr database to remove protein coding genes (E-value threshold of 10).

### miRNAs prediction using *C. robusta* small RNA-Seq data

The *C. robusta* small RNA-Seq reads (SRR038843, SRR038844, 26 nt) were mapped onto the repeat-sequence-masked *H. roretzi* reference genome using BLASTN (E-value of ≤1000, word size of 10). Only the best hits with a high-scoring segment pair (HSP) identity of ≥90% and an alignment length of ≥20 and ≤2 mismatches were kept. Then, we examined possible stem-loop structures in the sequences and the flanking 100 bp sequences using srnaloop [38]. Sequences whose minimum folding free energy (MFE) and stem-loop structure passed our filtration criteria were reserved. Among the candidate stem-loops, those with more than ten *H. roretzi* mRNA reads in mRNA-seq results (C. Dantec, H. Nishida and P. Lemaire, unpublished results) were excluded.

### Filtration criteria

All the potential miRNAs reported in this study passed the following filtration criteria. The miRNA precursors were generally considered that they have a smaller minimum folding free energy (MFE, ΔG kcal/mol) than ordinary genomic sequences of the same length. In this study, the MFE of potential precursors were obtained by folding their sequences using RNAfold, and whose minimum folding free energy not satisfying the following threshold were excluded from further analysis:

MFE ≤ -0.31 * L + 6.00 (see Fig. 6c)

Where, L indicates the predicated precursor length. The relationship between MFE and the length of miRNA precursor were tested on all metazoan species and simulated by a linear regression analysis [53, 54]. Then, the stem-loop structures were manually checked to exclude sequences with big bulges, or in which a part of mature miRNA is presented in the loop. To reduce false positive predictions, precursors that contain more than one loop (folded by RNAfold) were discarded (see Fig. 6b).

The GC contents are relatively low in the *C. robusta* (34.7%) and *H. roretzi* (35.7%) genome sequences. We have calculated GC content of the miRNAs in *C. robusta* (see Fig. 6d, blue bars). The GC content is not as biased as that of the genome. Therefore, we used 30–70% GC contents as filtration criterion since most miRNAs of *C. robusta* also fit this criterion. There could be a constraint on the GC contents of miRNAs as they have to firmly bind to target mRNAs.

### Inference of Mature miRNA for novel miRNA precursors

Patscan were used to find 5p and 3p mature miRNAs. The pattern file is: p1 = 22…27 5…50 ~ p1[6,0,0]. “p1 = 22…27” means that match the 5p mature miRNA sequence whose length is between 22 nt and 27 nt. “5…50” means that the distance between 5p and 3p mature miRNAs could range from 5 nt to 50 nt. “ ~ p1[6,0,0]” means that match the 3p mature miRNA sequence by allowing up to six mismatches to the reverse and complement 5p mature miRNA sequence. If more than one pairs were detected, the one closest to loop structure was kept. If no paired sequence found, the 24 bp pair sequences closest to the loop structure would be used. Putative miRNAs and miRNA stars were simply named using the suffix “5p” and “3p” subsequently.

### Inference of strand of miRNA precursors

The strands of the miRNAs were determined according to the miRBase database (Release 21) [44] (best BLASTN hit), *Halocynthia* ESTs (http://magest.hgc.jp) (E-value ≤ 1e-5) and *Ciona* small RNA-Seq data (best BLASTN hit). In miRNAs without a significant hit, the sequences of them are tentatively represented by those in plus strand of the reference genome.

### Prediction of miRNA targets

To get insight into the functions of the miRNAs, 3′ UTR sequences (defined as 400 bp sequence that immediately follows the translation stop codon in the genome sequence in this study) were extracted from each gene model in Aniseed [19] (http://www.aniseed.cnrs.fr/). We adopted RNAhybrid [55] to identify miRNA targets with p-value ≤ 0.01. Considering that position of the putative mature miRNAs could not be precisely inferred, we adjusted the seed sequence by using a series of helix constraint parameter “-f 1,6”, “-f 2,7”, “-f 3,8”,“-f 4,9”, “-f 5,10”,“-f 6,11”.

### Validation of miRNAs via RT-PCR

To validate the expression of predicted miRNAs, embryonic samples of six stages, unfertilized egg, blastula, gastrula, neurula, tailbud and hatched larvae were collected. Eggs of two adults were fertilized with sperms of five adults. Follicle cells that reside outside of the vitelline membrane and test sells that reside inside of the vitelline membrane were removed by digesting the vitelline membrane with protease, as previously described [56]. Total RNAs were extracted using TRIzol Reagent (Life Technologies). The quantity of total RNA was examined using Ultrospec 2100 pro (Life Sciences) and RNA integrity was assessed via agarose gel electrophoresis. RT-PCR primers (Additional file 4) were synthesized and purchased from Eurofins Genomics (Tokyo, Japan). Simple miRNA Detection Kit (BioDynamics Laboratory Inc. Japan) was used for RT-PCR according to the manufacture’s protocol. The PCR products were exposed to UV light after the polyacrylamide gel electrophoresis, and the images were taken with NIPPON genetics FAS-IV illuminator. The expected size of the amplified fragment is ~40 bp because the 19 bp universal primer is amplified together with ~20 bp miRNA-specific primers. To validate sequences of the PCR products, they were dissected from the gel, subcloned into the pGEM-T Easy vector (Promega), and sequencing reaction was performed at Eurofins Genomics (Tokyo, Japan).

## Additional files

Additional file 1: List and information of predicted miRNAs of *H. roretzi*. (XLS 118 kb)

Additional file 2: Phylogenic survey of the conserved *H. roretzi* miRNAs in other species. This Figure is similar to Fig. 1, but all of species names are given in this. (PDF 61 kb)

Additional file 3: *Ciona robusta* (formerly *intestinalis*) miRNAs that are conserved across metazoans. (XLS 33 kb)

Additional file 4: Primers used to validate expression of the miRNAs. (DOC 48 kb)

Additional file 5: miRNAs associated with various development processes and their target GO annotation. (XLS 216 kb)

## Abbreviations

CM: Covariance model
GO: Gene ontology
HSP: High-scoring segment pair
MFE: Minimum folding free energy

## References

[1] Yu Z, Li Y, Fan H, Liu Z, Pestell RG (2012). miRNAs regulate stem cell self-renewal and differentiation. Front Genet.

[2] Nakano H, Yamada Y, Miyazawa T, Yoshida T (2013). Gain-of-function microRNA screens identify miR-193a regulating proliferation and apoptosis in epithelial ovarian cancer cells. Int J Oncol.

[3] Tzur G, Israel A, Levy A, Benjamin H, Meiri E, Shufaro Y, Meir K, Khvalevsky E, Spector Y, Rojansky N (2009). Comprehensive gene and microRNA expression profiling reveals a role for microRNAs in human liver development. PLoS One.

[4] Shiiba M, Shinozuka K, Saito K, Fushimi K, Kasamatsu A, Ogawara K, Uzawa K, Ito H, Takiguchi Y, Tanzawa H (2013). MicroRNA-125b regulates proliferation and radioresistance of oral squamous cell carcinoma. Br J Cancer.

[5] Martinez NJ, Walhout AJ (2009). The interplay between transcription factors and microRNAs in genome-scale regulatory networks. Bioessays.

[6] Lewis BP, Burge CB, Bartel DP (2005). Conserved seed pairing, often flanked by adenosines, indicates that thousands of human genes are microRNA targets. Cell.

[7] Li SC, Pan CY, Lin WC (2006). Bioinformatic discovery of microRNA precursors from human ESTs and introns. BMC Genomics.

[8] Rodriguez A, Griffiths-Jones S, Ashurst JL, Bradley A (2004). Identification of mammalian microRNA host genes and transcription units. Genome Res.

[9] Hao Y, Liu JR, Zhang Y, Yang PG, Feng YJ, Cui YJ, Yang CH, Gu XH (2016). The microRNA expression profile in porcine skeletal muscle is changed by constant heat stress. Anim Genet.

[10] Chen CC, Fu SF, Norikazu M, Yang YW, Liu YJ, Ikeo K, Gojobori T, Huang HJ (2015). Comparative miRNAs analysis of Two contrasting broccoli inbred lines with divergent head-forming capacity under temperature stress. BMC Genomics.

[11] Bedreag OH, Sandesc D, Chiriac SD, Rogobete AF, Cradigati AC, Sarandan M, Dumache R, Nartita R, Papurica M (2016). The Use of Circulating miRNAs as Biomarkers for Oxidative Stress in Critically Ill Polytrauma Patients. Clin Lab.

[12] Jian H, Wang J, Wang T, Wei L, Li J, Liu L (2016). Identification of Rapeseed MicroRNAs Involved in Early Stage Seed Germination under Salt and Drought Stresses. Front Plant Sci.

[13] Cai P, Hou N, Piao X, Liu S, Liu H, Yang F, Wang J, Jin Q, Wang H, Chen Q (2011). Profiles of small non-coding RNAs in Schistosoma japonicum during development. PLoS Negl Trop Dis.

[14] Xie L, Yang Z, Li G, Shen L, Xiang X, Liu X, Xu D, Xu L, Chen Y, Tian Z (2013). Genome-wide identification of bone metastasis-related microRNAs in lung adenocarcinoma by high-throughput sequencing. PLoS One.

[15] Liu N, Yang J, Guo S, Xu Y, Zhang M (2013). Genome-wide identification and comparative analysis of conserved and novel microRNAs in grafted watermelon by high-throughput sequencing. PLoS One.

[16] Wang T, Chen L, Zhao M, Tian Q, Zhang WH (2011). Identification of drought-responsive microRNAs in Medicago truncatula by genome-wide high-throughput sequencing. BMC Genomics.

[17] Lemaire P (2011). Evolutionary crossroads in developmental biology: the tunicates. Development.

[18] Satoh N (2001). Ascidian embryos as a model system to analyze expression and function of developmental genes. Differentiation.

[19] Brozovic M, Martin C, Dantec C, Dauga D, Mendez M, Simion P, Percher M, Laporte B, Scornavacca C, Di Gregorio A (2016). ANISEED 2015: a digital framework for the comparative developmental biology of ascidians. Nucleic Acids Res.

[20] Dehal P, Satou Y, Campbell RK, Chapman J, Degnan B, De Tomaso A, Davidson B, Di Gregorio A, Gelpke M, Goodstein DM (2002). The draft genome of Ciona intestinalis: insights into chordate and vertebrate origins. Science.

[21] Brunetti R, Gissi C, Pennati R, Caicci F, Gasparini F, Manni L (2015). Morphological evidence that the molecularly determined Ciona intestinalis type A and type B are different species: Ciona robusta and Ciona intestinalis. J Zool Syst Evol Res.

[22] Vinson JP, Jaffe DB, O’Neill K, Karlsson EK, Stange-Thomann N, Anderson S, Mesirov JP, Satoh N, Satou Y, Nusbaum C (2005). Assembly of polymorphic genomes: algorithms and application to Ciona savignyi. Genome Res.

[23] Voskoboynik A, Neff NF, Sahoo D, Newman AM, Pushkarev D, Koh W, Passarelli B, Fan HC, Mantalas GL, Palmeri KJ (2013). The genome sequence of the colonial chordate, Botryllus schlosseri. Elife.

[24] Gyoja F, Satou Y, Shin-i T, Kohara Y, Swalla BJ, Satoh N (2007). Analysis of large scale expression sequenced tags (ESTs) from the anural ascidian, Molgula tectiformis. Dev Biol.

[25] Seo HC, Kube M, Edvardsen RB, Jensen MF, Beck A, Spriet E, Gorsky G, Thompson EM, Lehrach H, Reinhardt R (2001). Miniature genome in the marine chordate Oikopleura dioica. Science.

[26] Danks G, Campsteijn C, Parida M, Butcher S, Doddapaneni H, Fu B, Petrin R, Metpally R, Lenhard B, Wincker P (2013). OikoBase: a genomics and developmental transcriptomics resource for the urochordate Oikopleura dioica. Nucleic Acids Res.

[27] Stolfi A, Lowe EK, Racioppi C, Ristoratore F, Brown CT, Swalla BJ, Christiaen L (2014). Divergent mechanisms regulate conserved cardiopharyngeal development and gene expression in distantly related ascidians. Elife.

[28] Tsagkogeorga G, Turon X, Galtier N, Douzery EJ, Delsuc F (2010). Accelerated evolutionary rate of housekeeping genes in tunicates. J Mol Evol.

[29] Norden-Krichmar TM, Holtz J, Pasquinelli AE, Gaasterland T (2007). Computational prediction and experimental validation of Ciona intestinalis microRNA genes. BMC Genomics.

[30] Shi W, Hendrix D, Levine M, Haley B (2009). A distinct class of small RNAs arises from pre-miRNA-proximal regions in a simple chordate. Nat Struct Mol Biol.

[31] Keshavan R, Virata M, Keshavan A, Zeller RW (2010). Computational identification of Ciona intestinalis microRNAs. Zoolog Sci.

[32] Hendrix D, Levine M, Shi W (2010). miRTRAP, a computational method for the systematic identification of miRNAs from high throughput sequencing data. Genome Biol.

[33] Kusakabe R, Tani S, Nishitsuji K, Shindo M, Okamura K, Miyamoto Y, Nakai K, Suzuki Y, Kusakabe TG, Inoue K (2013). Characterization of the compact bicistronic microRNA precursor, miR-1/miR-133, expressed specifically in Ciona muscle tissues. Gene Expr Patterns.

[34] Chen JS, Pedro MS, Zeller RW (2011). miR-124 function during Ciona intestinalis neuronal development includes extensive interaction with the Notch signaling pathway. Development.

[35] Joyce Tang W, Chen JS, Zeller RW (2013). Transcriptional regulation of the peripheral nervous system in Ciona intestinalis. Dev Biol.

[36] Kozomara A, Griffiths-Jones S (2014). miRBase: annotating high confidence microRNAs using deep sequencing data. Nucleic Acids Res.

[37] Fu X, Adamski M, Thompson EM (2008). Altered miRNA repertoire in the simplified chordate, Oikopleura dioica. Mol Biol Evol.

[38] Grad Y, Aach J, Hayes GD, Reinhart BJ, Church GM, Ruvkun G, Kim J (2003). Computational and experimental identification of C. elegans microRNAs. Mol Cell.

[39] Wang C, Han J, Liu C, Kibet KN, Kayesh E, Shangguan L, Li X, Fang J (2012). Identification of microRNAs from Amur grape (Vitis amurensis Rupr.) by deep sequencing and analysis of microRNA variations with bioinformatics. BMC Genomics.

[40] Fu L, Niu B, Zhu Z, Wu S, Li W (2012). CD-HIT: accelerated for clustering the next-generation sequencing data. Bioinformatics.

[41] Dsouza M, Larsen N, Overbeek R (1997). Searching for patterns in genomic data. Trends Genet.

[42] Hatakeyama Y, Shibuya N, Nishiyama T, Nakashima N (2004). Structural variant of the intergenic internal ribosome entry site elements in dicistroviruses and computational search for their counterparts. RNA.

[43] Ryvkin P, Leung YY, Ungar LH, Gregory BD, Wang LS (2014). Using machine learning and high-throughput RNA sequencing to classify the precursors of small non-coding RNAs. Methods.

[44] Kozomara A, Griffiths-Jones S (2011). miRBase: integrating microRNA annotation and deep-sequencing data. Nucleic Acids Res.

[45] Li A, Mao L (2007). Evolution of plant microRNA gene families. Cell Res.

[46] Benson G (1999). Tandem repeats finder: a program to analyze DNA sequences. Nucleic Acids Res.

[47] Gardner PP, Daub J, Tate J, Moore BL, Osuch IH, Griffiths-Jones S, Finn RD, Nawrocki EP, Kolbe DL, Eddy SR (2011). Rfam: Wikipedia, clans and the “decimal” release. Nucleic Acids Res.

[48] Nawrocki EP, Kolbe DL, Eddy SR (2009). Infernal 1.0: inference of RNA alignments. Bioinformatics.

[49] Lowe TM, Eddy SR (1997). tRNAscan-SE: a program for improved detection of transfer RNA genes in genomic sequence. Nucleic Acids Res.

[50] Bompfunewerer AF, Backofen R, Bernhart SH, Hertel J, Hofacker IL, Stadler PF, Will S (2008). Variations on RNA folding and alignment: lessons from Benasque. J Math Biol.

[51] Gruber AR, Lorenz R, Bernhart SH, Neubock R, Hofacker IL (2008). The Vienna RNA websuite. Nucleic Acids Res.

[52] Gardner PP, Daub J, Tate JG, Nawrocki EP, Kolbe DL, Lindgreen S, Wilkinson AC, Finn RD, Griffiths-Jones S, Eddy SR (2009). Rfam: updates to the RNA families database. Nucleic Acids Res.

[53] Zhang BH, Pan XP, Cox SB, Cobb GP, Anderson TA (2006). Evidence that miRNAs are different from other RNAs. Cell Mol Life Sci.

[54] Adai A, Johnson C, Mlotshwa S, Archer-Evans S, Manocha V, Vance V, Sundaresan V (2005). Computational prediction of miRNAs in Arabidopsis thaliana. Genome Res.

[55] Rehmsmeier M, Steffen P, Hochsmann M, Giegerich R (2004). Fast and effective prediction of microRNA/target duplexes. RNA.

[56] Nishide K, Mugitani M, Kumano G, Nishida H (2012). Neurula rotation determines left-right asymmetry in ascidian tadpole larvae. Development.

