# Supplementary material for: Genome-wide survey of miRNAs and their evolutionary history in the ascidian, Halocynthia roretzi
Source: BMC Genomics. 2017 Apr 20;18:314. doi: 10.1186/s12864-017-3707-5 (PMC5399378; doi:10.1186/s12864-017-3707-5)
Supplement: Supplementary file 2 — Phylogenic survey of the conserved H. roretzi miRNAs in other species. This Figure is similar to Fig. 1, but all of species names are given in this. (PDF 61 kb) [file 12864_2017_3707_MOESM2_ESM.pdf]

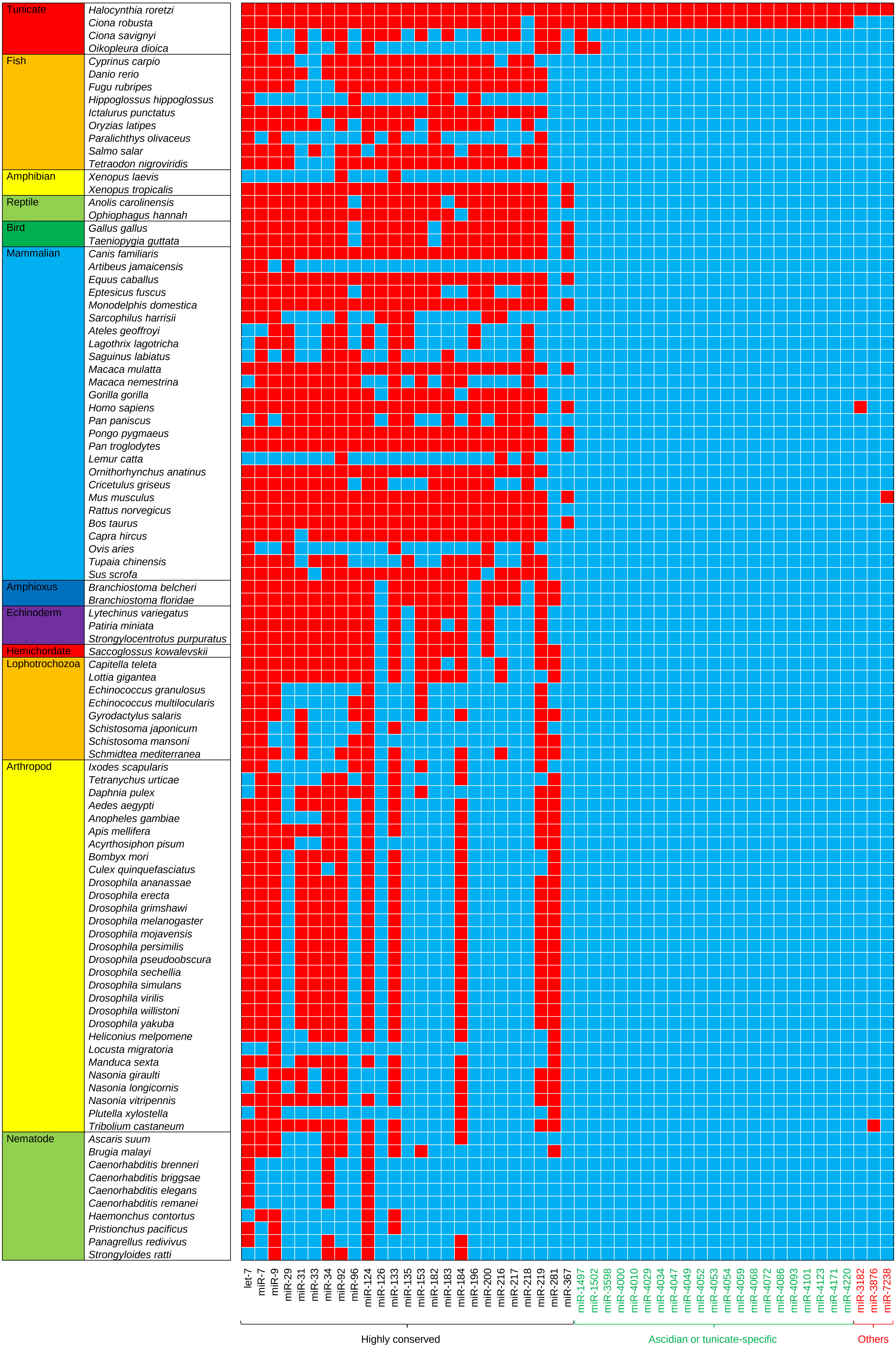
Tunicate
Halocynthia roretzi
Ciona robusta
Ciona savignyi
Oikopleura dioica
Fish
Cyprinus carpio
Danio rerio
Fugu rubripes
Hippoglossus hippoglossus
Ictalurus punctatus
Oryzias latipes
Paralichthys olivaceus
Salmo salar
Tetraodon nigroviridis
Amphibian
Xenopus laevis
Xenopus tropicalis
Reptile
Anolis carolinensis
Ophiophagus hannah
Bird
Gallus gallus
Taeniopygia guttata
Mammalian
Canis familiaris
Artibeus jamaicensis
Equus caballus
Eptesicus fuscus
Monodelphis domestica
Sarcophilus harrisii
Ateles geoffroyi
Lagothrix lagotricha
Saguinus labiatus
Macaca mulatta
Macaca nemestrina
Gorilla gorilla
Homo sapiens
Pan paniscus
Pongo pygmaeus
Pan troglodytes
Lemur catta
Ornithorhynchus anatinus
Cricetulus griseus
Mus musculus
Rattus norvegicus
Bos taurus
Capra hircus
Ovis aries
Tupaia chinensis
Sus scrofa
Amphioxus
Branchiostoma belcheri
Branchiostoma floridae
Echinoderm
Lytechinus variegatus
Patiria miniata
Strongylocentrotus purpuratus
Hemichordate
Saccoglossus kowalevskii
Lophotrochozoa
Capitella teleta
Lottia gigantea
Echinococcus granulosus
Echinococcus multilocularis
Gyrodactylus salaris
Schistosoma japonicum
Schistosoma mansoni
Schmidtea mediterranea
Arthropod
Ixodes scapularis
Tetranychus urticae
Daphnia pulex
Aedes aegypti
Anopheles gambiae
Apis mellifera
Acyrthosiphon pisum
Bombyx mori
Culex quinquefasciatus
Drosophila ananassae
Drosophila erecta
Drosophila grimshawi
Drosophila melanogaster
Drosophila mojavensis
Drosophila persimilis
Drosophila pseudoobscura
Drosophila sechellia
Drosophila simulans
Drosophila virilis
Drosophila willistoni
Drosophila yakuba
Heliconius melpomene
Locusta migratoria
Manduca sexta
Nasonia giraulti
Nasonia longicornis
Nasonia vitripennis
Plutella xylostella
Tribolium castaneum
Nematode
Ascaris suum
Brugia malayi
Caenorhabditis brenneri
Caenorhabditis briggsae
Caenorhabditis elegans
Caenorhabditis remanei
Haemonchus contortus
Pristionchus pacificus
Panagrellus redivivus
Strongyloides ratti
let-7
miR-7
miR-9
miR-29
miR-31
miR-33
miR-34
miR-92
miR-96
miR-124
miR-126
miR-133
miR-135
miR-153
miR-182
miR-183
miR-184
miR-196
miR-200
miR-216
miR-217
miR-218
miR-219
miR-281
miR-367
miR-1497
miR-1502
miR-3598
miR-4000
miR-4010
miR-4029
miR-4034
miR-4047
miR-4049
miR-4052
miR-4053
miR-4054
miR-4059
miR-4068
miR-4072
miR-4086
miR-4093
miR-4101
miR-4123
miR-4171
miR-4220
miR-3182
miR-3876
miR-7238
Highly conserved
Ascidian or tunicate-specific
Others
