# Supplementary material for: Genome-wide survey of miRNAs and their evolutionary history in the ascidian, Halocynthia roretzi
Source: BMC Genomics. 2017 Apr 20;18:314. doi: 10.1186/s12864-017-3707-5 (PMC5399378; doi:10.1186/s12864-017-3707-5)
Supplement: Supplementary file 4 — Primers used to validate expression of the miRNAs. (DOC 48 kb) [file 12864_2017_3707_MOESM4_ESM.doc]

**Additional file 4. Primers used to validate the expression of the microRNAs.**

| No. | MicroRNAs | Forward primers (5'-3') | Primer length (bp) | Tm (°C) | GC (%) |
| --- | --- | --- | --- | --- | --- |
| 0 | Universal Reveres Primer | GGTGCATTGATTCCCGAGT | 19 | 60.6 | 52 |
| 1 | miR-31 | TATGGCAAGATGTTGGCATAGC | 22 | 61.6 | 45 |
| 2 | miR-92a | TATTGCACTTGTCCCGGCC | 19 | 63.1 | 57 |
| 3 | let-7b | TGAGGTAGTAGGTTATATCAGT | 22 | 55.1 | 36 |
| 4 | miR-153 | TTGCATAGTAACAAAAGTGATC | 22 | 55.2 | 31 |
| 5 | miR-200 | TAATACTGCTTGGTAATGATG | 21 | 53.6 | 33 |
| 6 | miR-96 | TTTGGCACTAGCACATTATT | 20 | 55.8 | 35 |
| 7 | miR-184 | TGGACGGAGAATTGATAAGG | 20 | 57.2 | 45 |
| 8 | miR-196 | TAGGTAGTTACAAGTTGTGG | 20 | 54.5 | 40 |
| 9 | miR-281 | TGTCATGGAGTTGCTCTCTTATT | 23 | 60 | 39 |
| 10 | miR-367 | TATTGCACATTGTAATGGT | 19 | 52.5 | 31 |
| 11 | miR-5001 | AGTACCATCACGCGCCATGCC | 21 | 68.2 | 61 |
| 12 | miR-5003 | TTGAGCAATATCAGGATGTGCTG | 23 | 61.5 | 43 |
| 13 | miR-5008 | AACACCTTAATCACCAATGTG | 21 | 56.9 | 38 |
| 14 | miR-5004 | ATGGATTTGAGTAACCTGTAG | 21 | 55.2 | 38 |
| 15 | miR-5014 | TTTAATATCATGAGGGGGAGC | 21 | 57.4 | 42 |
| 16 | miR-124c | TATTAAGGCACGCGGTGAATGCC | 23 | 66.3 | 52 |
| 17 | miR-5070 | GAGGCGCGACGCGAAATGG | 19 | 67.5 | 68 |
| 18 | miR-4034 | GCACAGTGCTACAGGAAAG | 19 | 58.9 | 52 |
| 19 | miR-4029 | AAAGTGCAACAGTGTAAACC | 20 | 57.2 | 40 |
| 20 | miR-4123 | TGCTTCTTCCTCAGCTCGC | 19 | 62.8 | 57 |

Note: Universal primer of Simple miRNA Detection Kit (BioDynamics Laboratory Inc. Japan) was used for the reverse primer.
